# Targeting the MYCN interaction network in neuroblastoma

**DOI:** 10.1042/BSR20250104

**Published:** 2026-06-15

**Authors:** Chelsea Xinyi Yow, Sharon Yeoh, Eoin Leen, Richard Bayliss

**Affiliations:** 1 School of Molecular and Cellular Biology, Faculty of Biological Sciences, University of Leeds, LS2 9JT, U.K.; 2 Astbury Centre for Structural Molecular Biology, University of Leeds, LS2 9JT, U.K.

**Keywords:** degraders, MYCN amplification, neuroblastoma, protein-protein interactions

## Abstract

Neuroblastoma is a common solid tumour in children and accounts for a disproportionate share of childhood cancer mortality. High-risk neuroblastoma is a major clinical challenge, with survival rates below 50% despite intensive therapy. Amplification of the *MYCN* oncogene is a hallmark of high-risk disease. MYCN protein is a transcription factor that promotes proliferation and blocks differentiation. MYCN is often described as ‘undruggable’ due to its lack of enzymatic activity and intrinsically disordered nature that means it lacks well-defined binding pockets for small molecules. This review explores the molecular interactome of MYCN as an opportunity for therapeutic discovery. We highlight functional and structural features of the interactions with key partners, such as MAX, WDR5, TFIIIC5 and Aurora kinase A. We discuss emerging strategies to disrupt MYCN interactions and to enable its degradation through disruption of protein stabilisation mechanisms or the use of degraders such as proteolysis targeting chimeras. By integrating knowledge of MYCN biology, molecular structures and chemical biology, these approaches provide promising routes towards targeted therapies for *MYCN*-driven neuroblastoma.

## Neuroblastoma

Neuroblastoma is an embryonal tumour of the sympathetic nervous system, which arises from primordial neural crest cells (NCCs) and generally results in tumours in the adrenal glands or the sympathetic ganglia [1]. It is a heterogeneous disease in which tumours can spontaneously degenerate or develop, meaning that some neuroblastomas resolve without reason while some mature and spread rapidly [2]. This heterogeneity of neuroblastoma clinical presentation—ranging from asymptomatic benign tumours to aggressive metastases—leads to different prognosis and outcomes [3].

Neuroblastoma is the most common solid extracranial tumour in infants and children, constituting 6%–10% of malignancies diagnosed in paediatric patients. However, it accounts for a disproportionately high percentage of cancer-associated deaths in children at ∼15% [4]. Around 90% of neuroblastomas were diagnosed in children younger than 5 years old, with approximately 30% of these cases reported within the first year, and nearly all cases of neuroblastoma were diagnosed before 10 years of age [5,6]. The median age of neuroblastoma diagnosis is 17–22 months; it is rarely diagnosed in adolescents and adults but, if so, clinical trajectories and prognosis are much poorer for this age group [7,8].

Considering the variability of outcomes, the International Neuroblastoma Staging System (INSS) was designed to stratify patients into groups: low-risk, intermediate-risk, or high-risk. Risk levels are dependent on the patient’s INSS stage, age at diagnosis, tumour histopathology, DNA index, and *MYCN* amplification status [9]. However, the scale of surgical resection was taken into consideration in this staging system, which is problematic as it prohibits pretreatment stage classification, thereby making it complicated and non-standardised [10]. Hence, a more specific system called the International Neuroblastoma Risk Group Staging System was constructed to better categorise patients based on clinical presentation and tumour biology to determine further therapeutic approaches [11].

## Symptoms of neuroblastoma

**Figure 1:**
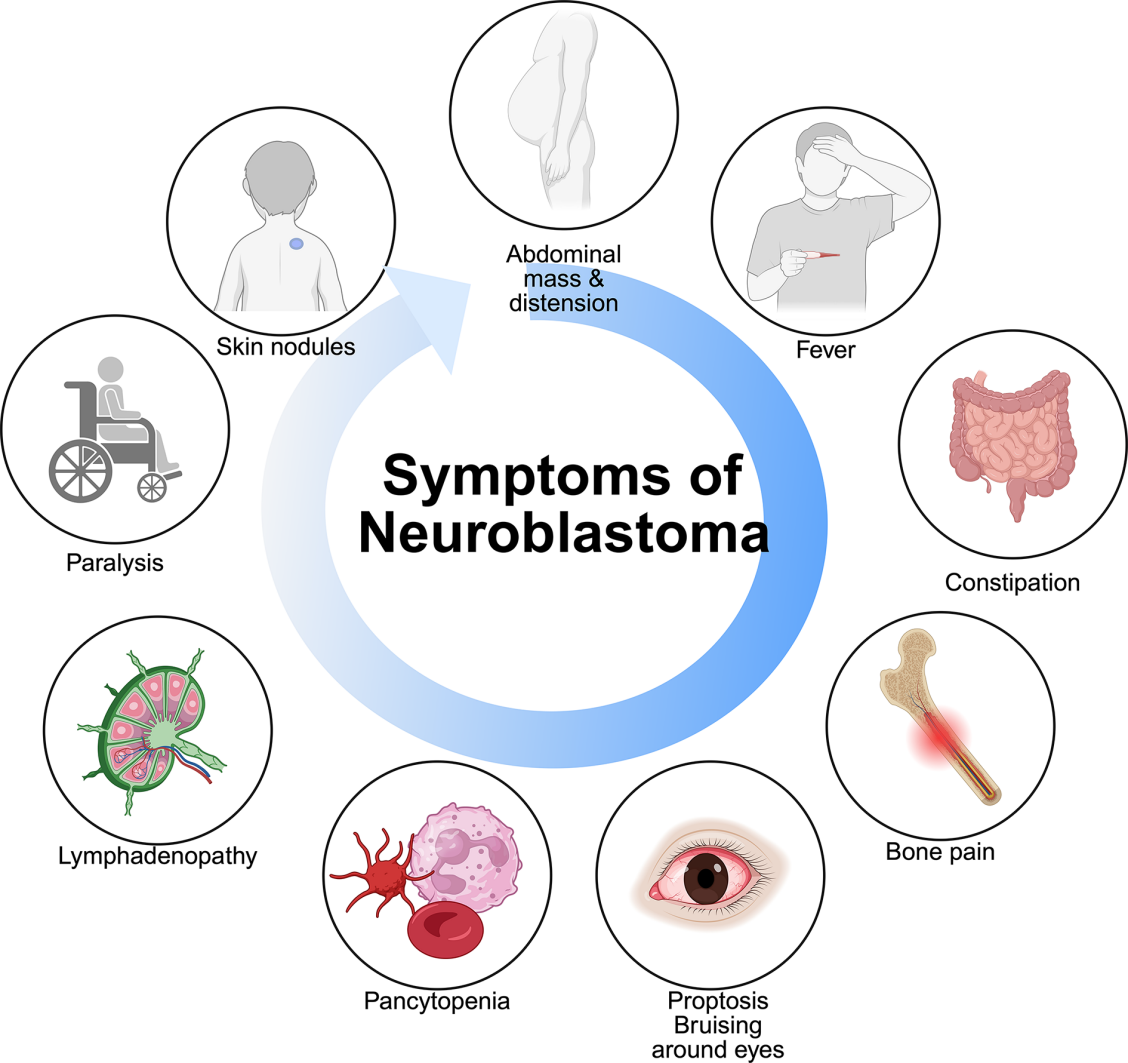
Overview of neuroblastoma symptoms The symptoms of neuroblastoma are diverse and affect different parts of the body. The symptoms are illustrated in order from more common (darker blue) to less common (lighter blue). Created in BioRender; Bayliss, R. (2026) https://BioRender.com/pxrk4mi

Neuroblastoma tumours can develop in areas where sympathetic nervous tissues are present [8]. The most common primary tumour site in children is the abdomen, which predominantly affects the adrenal glands, making abdominal mass the most typical symptom of neuroblastoma [8,12]. However, tumours can manifest anywhere along the sympathetic chain, this includes the neck, chest, and pelvis [8]. Up to 60%–70% of neuroblastoma patients exhibit metastatic disease that spreads via both lymphatic vessels and the bloodstream to sites such as the bone marrow, bone, skin, liver, and lymph nodes [13]. Metastases to these sites cause directly linked symptoms such as pancytopenia, bone pain, subcutaneous skin nodules, abdominal distention, and lymphadenopathy [10]. Other symptoms include proptosis, paralysis, diarrhoea, Horner syndrome, fever, and so forth. In rare cases (<5%), neuroblastoma metastasise to the central nervous system and lungs [14]. This illustrates the breadth of neuroblastoma clinical presentation, highlighting the difficulty of accurate diagnosis especially when patients present atypical manifestations (Figure 1).

## Low- versus high-risk neuroblastoma

Low- and intermediate-risk patients have approximately 80%–95% event-free survival rate whereas high-risk patients have less than 50% chance of event-free survival, and there is a further group of refractory high-risk patients that are unresponsive to therapy [15]. Treatment is highly dependent on the risk group of the patient; low- and intermediate-risk patients require less aggressive therapy such as surgery, short-term chemotherapy, or radiation therapy [10]. Children with high-risk neuroblastoma require three phases of rigorous and intensive treatment that generally includes: surgery, chemotherapy, radiation, stem cell transplant, immunotherapy, and retinoid therapy [10].

Despite significant improvement in 5-year survival rates from 29% to 50% in the past 20 years, patients with high-risk neuroblastoma still exhibited poor prognosis and have a heightened likelihood of relapse [16,17]. Patients that suffer from relapsed neuroblastoma are rarely cured. This underscores the sharp disparity between low- and high-risk neuroblastoma in terms of treatment approaches and prognosis, emphasising the urgency for effective and long-lasting therapeutic interventions against high-risk neuroblastoma.

Among patients with high-risk neuroblastoma, *MYCN* amplification was found to be a driver of aggressive disease progression, making it one of the most powerful markers of poor prognosis [18]. This demonstrates the relationship between *MYCN* amplification and the progression to high-risk neuroblastoma. Hence, targeting and inhibiting *MYCN* amplification may provide a strategy to achieve better therapeutic outcomes for high-risk neuroblastoma [17].

## MYCN and MYC

MYC proteins are a family of transcription factors comprising three paralogs *MYC*, *MYCL*, and *MYCN*. They have physiological functions in development and tissue homeostasis [19]. These proteins become oncogenic through a plethora of mechanisms, including gene amplification, chromosomal translocation, enhancer dysregulation, signalling-driven activation, protein stabilisation and, rarely, point mutations [20,21]. Of the samples investigated in the Pan-Cancer Atlas (<9000 samples covering 33 tumour types) 28% had *a MYC* paralog amplification [22]. The oncogenic functions of MYC proteins are thought to be primarily, though not exclusively via amplification of existing transcriptional programs [23–25]. This can involve activation or repression of target genes that are required for diverse biological processes, such as proliferation, metabolism, differentiation, apoptosis, resulting in aberrant dysregulation of these processes [19,26,27].

The fundamental nature of MYC function is conserved through evolution, as most clearly demonstrated through studies on the cnidarian Hydra [28]. The structure and biological functions of *MYC* and *MYCN* are similar; they are highly homologous, and encode for gene products of similar sizes that share conserved regions for DNA–protein and protein–protein interactions [29]. However, *MYC* and *MYCN* differ in their spatiotemporal expression. Studies on the three mouse *MYC* paralogs show that while *MYCN* and *MYCL* have specialised roles in restricted tissues and developmental stages, *MYC* has a more generalised role [30]. Homozygous deletion of either *MYC* or *MYCN* in mouse embryonic stem cells did not result in uncontrolled proliferation or differentiation, indicating redundant functions [29]. However, the redundancy is incomplete because although replacement of the endogenous *MYC* gene with *MYCN* rescued embryonic lethality, and therefore most critical biological functions, the mice were smaller and had other defects [31].

## The role of MYCN in neuroblastoma

While *MYC* is implicated in various cancer types, *MYCN* has a much more limited and specific role—driving paediatric malignancies originating from the central and peripheral nervous system to cause neuroblastoma, retinoblastoma, glioblastoma, and medulloblastoma [32]. *MYCN* is expressed in specific cell lineages during development with minimal expression in normal paediatric and adult tissues, hence, targeting *MYCN* may have a favourable therapeutic window [33]. However, many current approaches rely on indirect targeting of MYCN.

*MYCN* is located on the distal short arm of chromosome 2 (2p24) and this region is amplified in a subset of neuroblastoma patients [34]. *MYCN* amplification, which is commonly defined as greater than 10 copies of gene per cell, is observed in approximately 25% of patients with primary neuroblastoma and a substantial fraction, but not the majority, of high-risk cases [34]. *MYCN* amplification is a result of genomic instability and arises randomly. The additional copies of *MYCN* are located in individual circular extrachromosomal DNA molecules (ecDNA, also called double minute chromatin bodies) and self-repeating arrays on a chromosome called homogenously staining regions [35,36]. It was initially thought that other genes might be co-amplified with *MYCN*, however, only *MYCN* is consistently amplified across different tumour samples from an individual, and other genes such as ALK are occasionally co-amplified [37–39]. Crucially, however, enhancer sequences are co-amplified with the MYCN gene [40]. The enhancers are recognised by core regulatory circuit (CRC) transcription factors that drive expression of each copy of the MYCN gene, and which reinforce the expression of other CRC factors. Thus, MYCN expression is sustained at a high level in neuroblastoma cells.

*MYCN* plays a multifaceted role in the pathogenesis of neuroblastoma; it induces malignancy and maintains the stem-like state of NCCs, and it stimulates proliferation and prevents apoptosis of NCCs that contribute to neuroblastoma formation [41,42]. As a transcription factor, the *MYCN* oncogene fuels the survival of cancer cells by activating genes associated with cancer hallmarks such as genes that increase proliferation, metastasis, pluripotency, self-renewal [29]. Concurrently, it suppresses genes that antagonise tumorigenesis to sustain stemness, bypass cell cycle arrest, and evade immune surveillance [29].

Another key function of *MYCN* in neuroblastoma lies in its ability to suppress terminal differentiation of sympathetic neurons (neural crest-derived cells) [43]. In normal NCCs, differentiation is tightly modulated throughout embryonic development by complex gene regulatory networks. *MYCN* is expressed at high levels that drives proliferation and thus migration of NCCs from the neural plate border to the ventral region in early stages of CNS development [44]. Subsequently, *MYCN* expression levels decreases, which is critical for NCCs to undergo differentiation into other NCC-derived cell types [45]. For NCC-derived lineages that function in maintaining pluripotency and self-renewal, *MYCN* remains at high levels [46]. In neuroblastoma, dysregulated differentiation and migration is observed in neuroblasts (a type of NC-derived cells) due to *MYCN* amplification [43]. Asymmetrical cell division is a physiological process where self-renewal and differentiation is precisely balanced; but in neuroblastoma, overexpression of *MYCN* promotes symmetric cell division that increases self-renewal and expands the undifferentiated cell population [47,48]. This sheds light on the role of *MYCN* in not only tumour advancement, but also tumour initiation.

## MYCN structure and interactome

**Figure 2:**
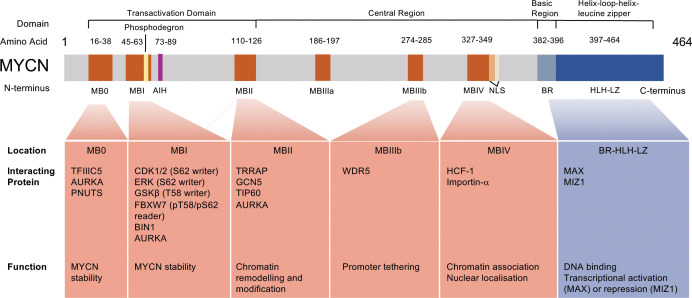
Structural features and functional domains of MYCN and their interactions with protein partners The functional domains of MYCN include: the transactivation domain (TAD), central region domain, DNA binding domain, and the dimerisation domain. The orange boxes show the highly conserved MBs. Within MBI, the T58 and S62 phosphorylation sites are located within a phosphodegron (yellow box). The Aurora kinase A (AURKA)-interacting helix (AIH, purple) is specific to MYCN. The light-yellow box is the nuclear localisation signal (NLS) that overlaps with MBIV. The light blue box denotes the basic region (BR) and the dark blue denotes the helix-loop-helix-leucine-zipper (HLH-LZ) domain. The amino acids for the boundaries of structural and functional domains are indicated above. Examples of MYCN-interacting proteins, the specific regions of *MYCN* involved in these interactions, and functions are included below.

Human MYCN and MYC proteins have around 45% sequence identity, consistent with a high density of conserved functional sites. Indeed, these conserved residues cluster in the key sites of protein–protein interactions, as discovered in interactome mapping studies [49,50]. MYCN comprises 464 amino acids with a molecular weight of approximately 50 kDa and contains six short sequences called MYC homology boxes (MBs): MB0, MBI, MBII, MBIIIa, MBIIIb, and MBIV that are highly conserved in MYC and MYCN (Figure 2). [51]. Each MB motif contributes to macromolecular interactions, many of which are associated with transcriptional regulation, chromatin remodelling, and phospho-regulation of stability [50].

MB0, MBI, and MBII are located within the TAD at the N-terminus of MYCN. MB0 is not necessary for tumour initiation, instead, it accelerates tumour growth and, at a molecular level, it facilitates interactions with the general transcriptional elongation factors TFIIF and TFIIIC [50,52]. MB0 also interacts with the kinase AURKA and the protein phosphatase 1-associated protein PNUTS [53,54]. Residues T58 and S62 within the phosphodegron in MBI are important phosphorylation sites for the regulation of MYCN activity and stability during the cell cycle [55,56]. MBII is crucial for transformation and transcriptional activation, mediated through the conversion of chromatin from a repressed to an active state, through the assembly of histone acetyltransferase complexes (HATs). The scaffold protein TRRAP and its associated HAT complexes are key interaction partners of MBII [50]. These include the GCN5-containing STAGA complex that contributes to the chromatin localisation and transcriptional activity of MYCN [57]. Remarkably, the co-expression of non-transforming MB0 (ΔMB0) and MBII deletion (ΔMBII) resulted in rescue of the transforming activity of *MYCN* to accelerate tumour growth. This trans-complementarity suggests that these myc boxes can act semi-autonomously with different binding partners to achieve a common goal—facilitating oncogenic *MYCN* activity [50].

The central region contains MBIIIa, MBIIIb, MBIV, and an NLS sequence (Figure 2). The NLS sequence overlaps with MBIV and is required for nuclear import of MYCN via importin-α, an interaction that has been resolved by X-ray crystallography [58]. MBIV interacts with HCF-1, a multi-domain transcriptional co-regulator that acts as a scaffold for transcription factors and chromatin-modifying enzymes [59]. The MBIIIb motif binds WDR5, a component of the COMPASS/MLL histone methyltransferase complex, which enables MYCN to bind to chromatin and maintain an active transcriptional state [60]. A crystal structure shows the molecular recognition of MYC MBIIIb by the β-propeller domain of WDR5, at a distinct site from its other interactors such as MLL [61].

The C-terminal region of MYCN comprises a BR for sequence-specific DNA binding tethered to an HLH-LZ domain that enables MYCN to interact with partner proteins that also contain the bHLH-LZ domain, such as MAX, to form heterodimers [62]. In the absence of DNA, the basic region can adopt a helical structure, but has greater conformational flexibility [63]. MYC can form alternative complexes with Miz1, a zinc-finger transcription factor, and thereby repress the transcription of cell cycle inhibitor genes [64]. However, MYCN interacts much more weakly with Miz1 than MYC, and this difference underlies distinct oncogenic programmes in MYC-driven versus MYCN-driven cancers [65].

MYCN is comprised of mainly intrinsically disordered regions (IDRs) with some regions of helical propensity [63,66,67]. Upon binding to partners or through post-translational modifications, these IDRs can undergo transition from disorder to order, or they can persist in a disordered state to form ‘fuzzy’ complexes [52,53,67,68]. MYCN may bind to its many partners in different stable conformations or in a dynamic state. Additionally, this implies that directly targeting MYCN will be challenging because of its structural dynamicity. Therefore, targeting these regions is difficult due to the lack of stability to form pockets for binding small molecules such as inhibitors or proteolysis targeting chimeras (PROTACs) [33].

In the following sections, we focus on common interactors for which structural models have been resolved, as these provide opportunities for structure-guided therapeutic discovery.

## MYCN and MAX

Mammals have a single *MAX* gene that can be differentially spliced to encode MAX protein variants. The two major forms of MAX are p21 and p22, which are constitutively expressed at high levels in quiescent, growing, and differentiating cells [69–71]. MAX, like MYCN, contains a bHLH-LZ region that enables specific dimerisation to form homodimers or heterodimers [70]. Studies *in vitro* found that, at equilibrium, MYCN/MAX heterodimers predominate over MYCN or MAX homodimers, therefore establishing a quantitative association between them [71–73]. MAX can also bind to other bHLH-LZ family members, however, each heterocomplex (with MYC and bHLH-LZ family members) has a distinct set of target genes [74].

**Figure 3:**
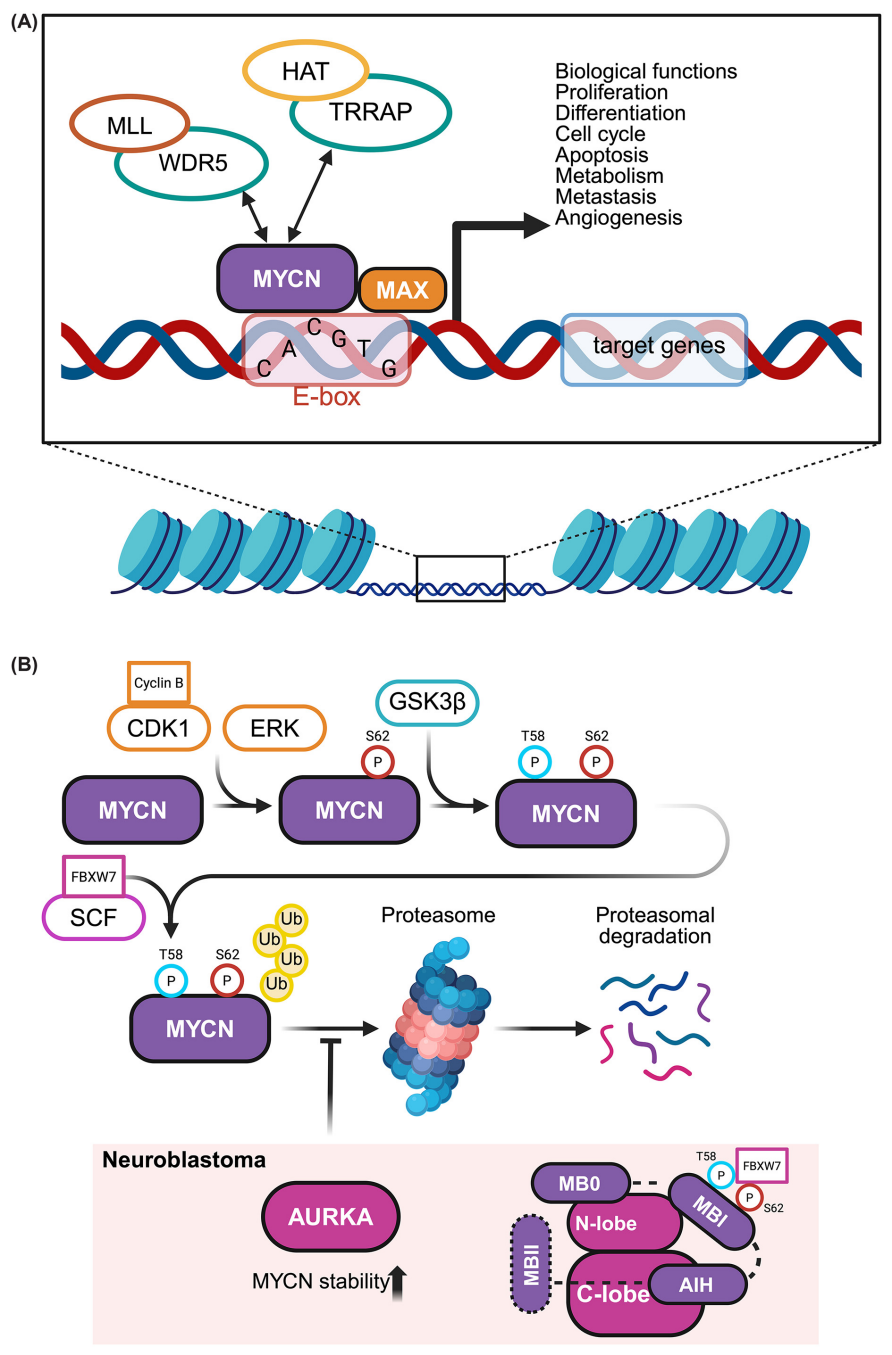
Molecular interactions of MYCN (**A**) MYCN and MAX form the heterodimer MYCN/MAX that binds to the specific DNA motif 5′-CACGTG-3′ (E-box). The complex then recruits proteins such as TRRAP/TIP60 and WDR5/MLL. These complexes function in chromatin remodelling and transcriptional regulation, keeping the chromatin uncondensed such that the target genes are transcriptionally active. The target genes of the MYCN/MAX heterodimer function in many pathways. (**B**) (Top section) In normal cells, the S62 and T58 residues in MYCN are phosphorylated sequentially. The phosphodegron serves as the recognition site of the FBXW7 ubiquitin ligase. FBXW7 then ubiquitinates MYCN, marking it for proteasomal degradation. (Bottom section) In neuroblastoma, AURKA is overexpressed and forms a complex with MYCN by binding to sites that flank its phosphodegron. This inhibits FBXW7-mediated proteasomal degradation, which increases MYCN stability. Specific regions of MYCN interact with either the N-lobe or C-lobe of AURKA. Binding site of MBII is not known. Created in BioRender; Bayliss, R. (2026) https://BioRender.com/i80c1oz

The MYCN/MAX heterodimer attaches preferentially to a binding motif (5′-CACGTG-3′) called the Enhancer-box (E-box) via the basic region of *MYCN* [75,76]. This complex recruits chromatin modifying enzymes to maintain the chromatin in an uncondensed state (Figure 3A). The heterodimer is crucial for MYCN function as without MAX, the protein is inactive and cannot regulate expression of target genes that are generally involved in key processes such as cell proliferation, growth, differentiation, and apoptosis [27,77]. In neuroblastoma, unlike normal cells, *MAX* expression is variable, and this is associated with clinical outcomes: high levels of MAX correlate with better outcomes when MYCN levels are low; but in *MYCN*-amplified neuroblastoma, high levels of MAX result in aggressive tumours [78]. MAX silencing in *MYCN*-amplified cells reduces their aggressiveness through reduced cell growth, motility, and promotion of cell differentiation [78].

## AURKA and MYCN stability

AURKA is a member of the mitotic serine/threonine kinase family. It is implicated in important mitotic processes such as the regulation of G2/M transition of the cell cycle, mitotic spindle assembly, and the maintenance of genetic fidelity [79]. Ordinarily, the expression of Aurora kinases is strictly cell-cycle-regulated: they are degraded in late mitosis, with only residual levels detected in the G1 phase [80]. AURKA is frequently dysregulated in cancer through gene amplification or impaired degradation, leading to elevated protein levels throughout the cell cycle [81]. Its aberrant persistence into G1 in cancer cells has been linked to increased proliferation and resistance to targeted therapies and chemotherapy [79].

In normal cells, MYCN is a short-lived protein with a half-life of approximately 30 minutes. MYCN is phosphorylated within MBI, which primes ubiquitinylation and proteolysis, thereby modulating protein stability [82]. Ser62 is first phosphorylated by kinases such as ERK or CDK1/cyclin B, and then Thr58 is phosphorylated by GSK3β (Figure 3B). Phosphorylation of these sites acts as the recognition site for SCF(FBXW7) ubiquitin ligase, which marks it for proteasomal degradation [83]. Although earlier work implicated further steps before FBXW7 binding, recent work has shown that the doubly phosphorylated form is recognised [84]. Interestingly, MYC has a second phosphodegron recognised by FBXW7, centred on phosphorylation of Thr244 and Thr248 [84]. However, this sequence is not conserved in MYCN, which is therefore dependent on the single phosphodegron in MBI.

In neuroblastoma, AURKA overexpression is associated with enhanced *MYCN* stability because it antagonises the recognition of the degradation signal by FBXW7 [85]. Consequently, MYCN is sequestered from degradation, which results in increased MYCN stability and accumulation within the cell. AURKA interacts with MYCN through an extensive interface, only part of which has been captured in a crystal structure [53]. This region of MYCN, the AIH, is C-terminal to the phosphodegron, interacts with the C-lobe of AURKA, which then competes with FBXW7. This motif lies outside an MYC box, and is not conserved in MYC, although the equivalent region has been captured in a crystal structure with the TBP/TAF1 complex, a core component of the TFIID complex that functions in RNA polymerase II initiation [86]. These equivalent motifs in MYC and MYCN both adopt helical structures when bound to their different partners, suggesting a degree of mechanistic similarity despite sequence differences. NMR studies have clarified that other regions of MYCN that interact with AURKA include MB0, MBI and MBII, and that the MB0-MBI region interacts with the kinase N-lobe [67,87]. NMR studies on MYC show a similar interaction pattern and, moreover, indicated that the interaction with AURKA is enhanced when MBI is phosphorylated [88]. Further studies are required to resolve the structural basis of AURKA interactions with MB0 and MBII at high resolution.

The AURKA–MYCN interaction is cell-cycle regulated and peaks during S-phase [49]. MYCN is also an activator of AURKA catalytic activity, and the recruitment of active AURKA to chromatin during S-phase helps to prevent transcription-replication conflicts through phosphorylation of substrates such as histone H3 [53,89]. Other binding partners of MYCN show an inverse cell-cycle dependence: they interact less strongly during S-phase. AURKA can directly displace factors from MYCN, including the TFIIIC complex.

## TFIIIC and MYCN chromatin localisation

TFIIIC is a six-subunit general transcription factor that binds to internal promoter elements (A-box, B-box) within Pol III-transcribed genes, such as tRNA genes [90]. After binding to the promoter, TFIIIC recruits TFIIIB, which positions RNA polymerase III at the transcription start site. TFIIIC also interacts with cohesin and CTCF, contributing to higher-order chromatin organisation beyond its pol III-specific role. TFIIIC modulates the localisation of MYCN on chromatin, but does not affect MYCN-dependent gene expression [91]. Instead, it appears to fine-tune an RNA-related function of MYCN: depletion of TFIIIC increases the occupation of active promoter hubs by MYCN, reducing the abundance of BRCA1 and nuclear exosome at these sites, key factors in promoter-proximal RNA degradation [92]. Recent structural studies resolved that MB0 of MYCN interacts with the DNA binding domain of TFIIIC5 [52]. Despite its name, this domain is not critical for specific DNA recognition by the TFIIIC complex. Instead, it provides a key interface with MYCN, the regulation of which is complicated by competition with non-specific DNA sequences and an intramolecular interaction with a C-terminal, acidic plug sequence in TFIIIC5. TFIIIC has thus emerged as a modulator of MYCN chromatin localisation and RNA surveillance activity, within a complex regulatory framework that may nonetheless provide opportunities for therapeutic intervention.

## Targeting MYCN as a therapeutic approach against neuroblastoma

Unlike many cancers, neuroblastoma has few mutations that restricts the number of potential targets for precision medicine. Many aspects of *MYCN* biology in neuroblastoma remain incompletely understood, including: the exact mechanisms of *MYCN* amplification, the regulation and roles of interactions between MYCN and its diverse protein partners, and which interactions underpin MYCN-driven tumour progression and poor clinical outcomes. These gaps limit the development of therapies that can specifically and effectively inhibit MYCN. Additionally, efforts to precisely target MYCN have proved to be challenging because of its dynamic, disordered nature, sparse structural data on MYCN complexes, and the difficulty of using traditional small molecules to block protein–protein or protein–DNA interactions [33]. Viewing MYCN as a network of protein complexes provides a framework for therapeutic targeting: disrupting chromatin engagement, rewriting transcriptional activity, or destabilising the protein.

## Chromatin association of MYCN

As MYCN functions as a transcription factor, the protein partners that mediate its interaction with chromatin represent potential targets to interrupt MYCN’s transcriptional activity. Most effort has been put towards developing inhibitors of the MYCN/MAX heterodimer. Small molecule inhibitors have been reported, such as 10074-G5, 10058-F4 and MYCi361 that bind MYC/MYCN and stabilise the intrinsically disordered monomer [93–95]. However, there remain challenges in developing small molecule MYC/MYCN–MAX compounds to be potent, selective, soluble and stable. Another approach has been to design peptide-based miniproteins, notably the pioneering work on Omomyc [96]. Omomyc is derived from the bHLH-LZ region of MYC, and it forms inactive heterodimers with MYC/MYCN and MAX to prevent MYC/MAX dimerisation. Omomyc has now been developed into a cell-penetrating version (OMO-103) that has safely completed phase I clinical trials in advanced, adult solid tumours [97].

Another promising approach is to target MYCN through its interaction with WDR5. WDR5 has two druggable sites: the WBM site that interacts with MYC/MYCN, and the WIN site that interacts with MLL, the catalytic subunit of the lysine methyltransferase for histone H3. Several WIN site inhibitors have been developed, such as OICR-9429 [98]. In contrast, the discovery of WBM site inhibitors has taken more time, in part due to the more challenging nature of the pocket [99–101]. Recent work has combined a WIN and WBM site inhibitor, with synergistic effects on neuroblastoma cell viability, and there are clear opportunities to explore the optimal targeting of WDR5 in neuroblastoma through drug combinations [101]. WDR5 inhibitors establish a paradigm that MYCN chromatin localisation and function can be disrupted without targeting its DNA binding domain. This concept will surely be explored in greater breadth in the coming years, through targeting other critical interaction partners of MYCN.

## Histone-modifying enzymes in MYCN transcriptional control

MYCN protein interacts functionally and physically with several histone-modifying enzymes that affect the transcriptional output of MYCN by regulating chromatin accessibility. Inhibiting these MYCN partners could indirectly inhibit the oncogenic transcriptional activity of MYCN through epigenetic modification or chromatin remodelling. These are enzymes with folded domains harbouring active sites and other pockets, and are therefore more straightforward targets for medicinal chemistry than the dynamic, disordered MYCN protein. However, this comes at the potential cost of a reduced therapeutic window due to other functions of these proteins.

There are clear functional links between MYCN and histone demethylases. MYCN recruits KDM4B to target genes, where it demethylates repressive H3K9me2/me3 marks [102]. Moreover, genetic deletion of any one of KDM4A, KDM4B, or KDM4C reduces the growth of MYCN-amplified neuroblastoma cells *in vitro* and in xenograft models [103]. Notably, the pan-KDM4 inhibitor QC6352 shows efficacy in mouse xenografts of two MYCN-amplified cell lines, and produces a durable response when combined with chemotherapy [103]. However, a comparable response is observed in a non-MYCN-amplified xenograft model, consistent with a broader programme of transcriptional control by KDM4 demethylases. The central region of MYCN and the PHD/Tudor domains of KDM4B are required for their association, although further work is required to map this more finely and to determine whether the interaction is direct [102].

**Figure 4:**
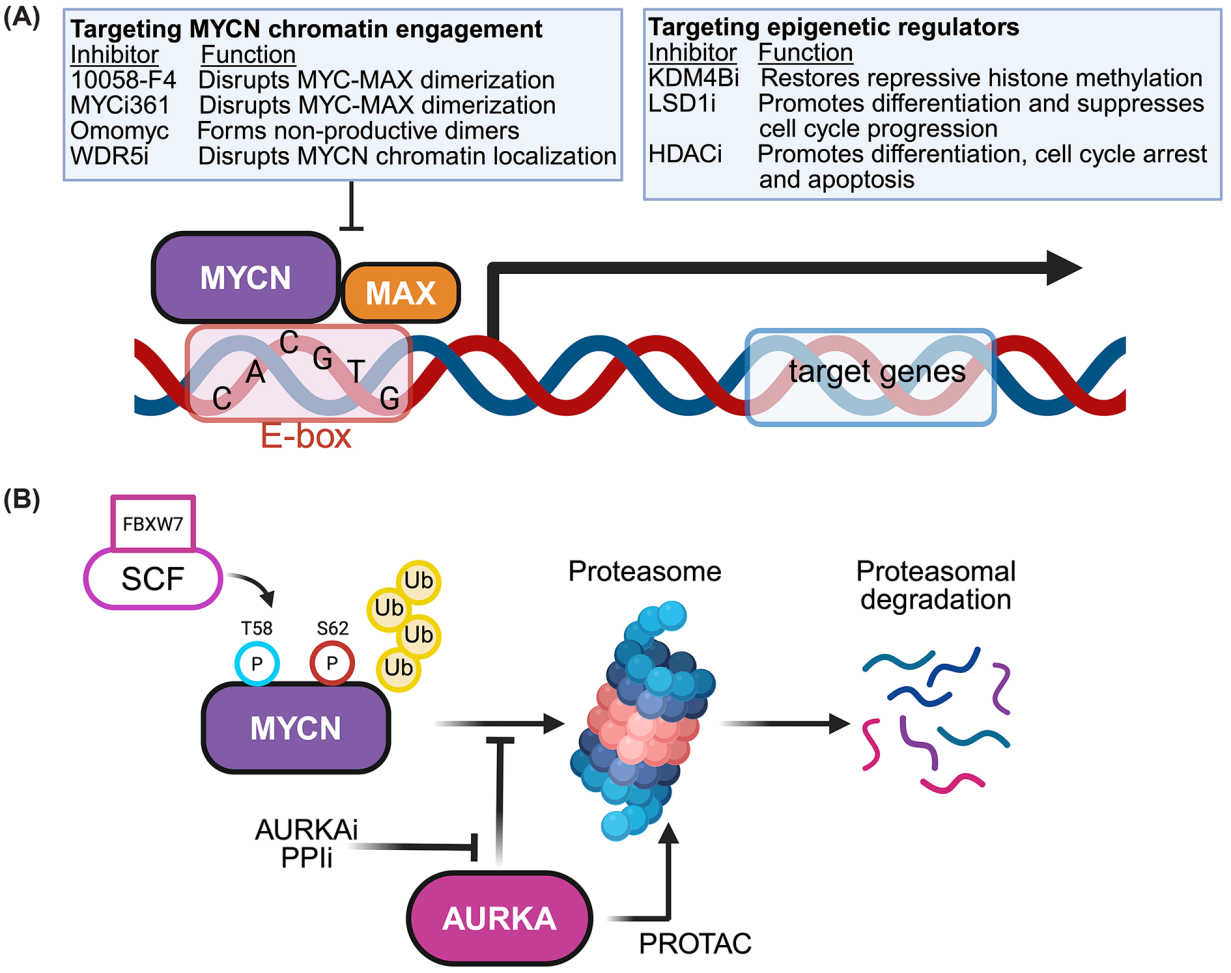
Potential therapeutic strategies targeting MYCN in neuroblastoma (**A**) Disrupt MYCN transcriptional via MYCN/MAX heterodimers or other factors. (**B**) Destabilise MYCN by targetting its interaction with AURKA either directly (PPIi) or by using a PROTAC that triggers AURKA degradation. Created in BioRender; Bayliss, R. (2026) https://BioRender.com/ay7s2eg

Along similar lines, LSD1 and HDAC1 are components of the CoREST co-repressor complex and candidate target histone-modifying enzymes in MYCN-amplified cancers (Figure 4A). LSD1, also called KDM1A, is a histone H3K4 and H3K9 demethylase with a critical role in silencing genes involved in neuronal differentiation. MYCN and LSD1 can be co-immunoprecipitated from neuroblastoma cells, an association that requires MBIII of MYCN [104]. MYCN and LSD1 co-localise to the promoter of the CDKN1A gene, which encodes the p21 cell-cycle inhibitor, and LSD1 inhibition relieves the suppression of its expression by MYCN [104]. There are numerous LSD1 inhibitors in clinical development, although neuroblastoma has not been the focus of attention. LSD1 inhibitors induce cell death in two different MYCN-amplified cell-lines, and the IMR32 cell-line that has higher LSD1 expression is more sensitive [105]. There is a need for more extensive, rigorous and mechanistic testing of LSD1 inhibitors in MYCN-amplified neuroblastoma.

There is substantial evidence that histone deacetylases (HDACs) play important roles in MYCN-driven transcriptional regulation of genes controlling cell cycle control, differentiation and apoptosis [106]. HDAC-containing complexes associate with MYCN at target gene promoters, which are likely mediated by multi-protein complexes, as there is no clear evidence for direct interactions [107]. These complexes regulate the landscape of cellular gene expression that determines cell fate. A complex comprising MYCN, SP1, and MIZ1 localises to TRKA and p75NTR promoters where recruitment of HDAC1 establishes a repressed chromatin state and suppresses NGF-induced apoptosis [107]. HDAC inhibition induces cell-cycle arrest in neuroblastoma cell models, for example by preventing entry into S-phase by downregulation of CDK4 and upregulation of p21 [108]. Because HDACs have pleiotropic functions, the focus of preclinical and clinical investigation has been on rational combination strategies to maximise therapeutic impact while minimising toxicity. For example, combining HDAC1/2 inhibitors with 13-*cis* retinoic acid, a standard component of maintenance for high-risk neuroblastoma, enhances differentiation and reduces viability in neuroblastoma cell lines [109]. However, these preclinical observations have not translated into robust clinical reponses. Further progress will require an improved understanding of the specific roles of individual HDACs in neuroblastoma biology, as well as a broader survey of rational combination therapy targets. For example, high-throughput screening has identified ubiquitin-specific protease 5 (USP5) inhibitors as synergistic with HDAC inhibition in MYCN-amplified neuroblastoma cell lines, whilst exhibiting low toxicity in normal human fibroblasts [110]. An additional priority is to elucidate the molecular mechanisms by which MYCN recruits HDAC-containing complexes to target promoters, and to develop inhibitors that selectively disrupt these interactions without affecting broader HDAC-dependent processes.

## Enabling MYCN degradation

Whilst there are many aspects of MYCN biology to be resolved, an attractive concept is to remove MYCN protein from neuroblastoma cells. This could be achieved by exploiting the mechanisms that confer stability on MYCN, such as its interaction with AURKA. Indirectly targeting MYCN through MYCN/AURKA interactions using certain AURKA inhibitors destabilises MYCN in neuroblastoma cell lines, which results in decreased MYCN protein levels and suppression of neuroblastoma tumour growth (Figure 4B). This works for certain AURKA inhibitors because they stabilise an inactive conformation of the kinase, such as MLN8054, MLN8237 (alisertib), and CD532, which are incompatible with the active kinase conformation recognised by MYCN [53,111–113]. However, clinical trials of alisertib exhibited significant haematological toxicity and an overall response rate of typically <15% [114,115]. There is clearly room for improvement, and the development of AURKA inhibitors that disrupt MYCN interaction continues, with recent examples including DBPR728 that shows activity against both MYC-high and MYCN-high cancer cell lines [116].

MYCN stimulates AURKA activity, which then phosphorylates histone H3S10 in S-phase, a function that could be exploited through combined inhibition of AURKA and ATR [89]. However, the mechanism by which AURKA stabilises MYCN degradation depends on a protein–protein interaction, and is independent of its kinase activity [53,85]. One future direction might be to develop inhibitors that block the interaction of AURKA with MYCN, without inhibiting the kinase to avoid toxicity associated with inhibiting the mitotic, kinase-dependent function of AURKA. This is challenging because the AURKA/MYCN interface has more than one site, and the only site resolved in a crystal structure overlaps with a key pocket for protein substate binding [53]. This problem might be resolved using peptidomimetic inhibitors, and tool molecules that disrupt this site directly or via an allosteric mechanism are at an early stage of development [117,118].

An emerging strategy for MYCN-driven neuroblastoma is the use of proteolysis targeting chimeras, bifunctional small molecules that recruit a protein target to a ubiquitin E3 ligase, enabling polyubiquitination and degradation. AURKA is an attractive PROTAC target because it is readily degradable and there are a plethora of inhibitors to serve as starting points [119]. AURKA PROTACs derived from alisertib have been described and, interestingly, they do not induce the severe mitotic arrest typical of alisertib or other AURKA kinase inhibitors [120,121]. This difference likely reflects partial protection of centrosome-associated Aurora-A, allowing the cells to progress through mitosis with only minor defects [121]. It is notable that alisertib-based PROTACs have not been reported to cause degradation of MYCN. This might be because alisertib stabilises an inactive conformation of AURKA that weakens the binding of MYCN, which prefers to bind an active kinase conformation [53]. While it makes sense to based PROTAC design on an inhibitor selective for AURKA, it might not be necessary because relatively unselective inhibitor scaffolds can form the basis of highly selective degraders. Indeed, a global map of kinase degradability placed AURKA as the second most degradable kinase, with AURKB outside the top twenty [119]. Therefore several further chemotypes have been explored to generate AURKA PROTACs:HLB-0532259 is based on the CDK4/6 inhibitor ribociclib [88]; SK2188, SK4454 and SK5527 are based on the AURKA inhibitor MK-5108 [122,123]. These PROTACs trigger rapid and selective degradation of AURKA and MYCN, suppress proliferation and induce apoptosis in MYCN- amplified neuroblastoma cells preferably compared to other cell types. This selectivity is consistent with selectivity of Aurora-A PROTACs for MYCN over MYC, but this has not been formally proven. It remains to be seen what is the optimal AURKA inhibitor from which to develop a PROTAC, but there are strong arguments in favour of Type I inhibitors that bind the active conformation of AURKA and favour ternary complex formation with MYCN [53,88].

Other MYCN interaction partners are being explored as PROTAC substrates, such as WDR5 and BET family proteins. Treatment of the human acute myeloid leukaemia cell-line MV4-11 with a WDR5-selective PROTAC MS67 reduced the chromatin association of MYC, while MYC protein levels were unaffected [124]. Although WDR5 PROTACs have not been tested in neuroblastoma cells, they can be expected to at least phenocopy, if not improve on WDR5–MYCN interaction disruptors. ARV-825, a PROTAC that induces the degradation of BET family proteins, required for high MYC/MYCN expression, shows activity in MYCN-driven neuroblastoma cells [125]. The PROTAC reduced MYCN protein levels indirectly, by reducing its expression, and was especially potent in reducing the cell viability of IMR-32, MYCN-high cells, although it is likely that factors other than MYCN contribute to this effect. There are currently no PROTACs that target MYCN itself, but a similar concept, molecular glue, has successfully been applied to MYC/MAX [126]. This compound, WBC100, glues the E3 ligase CHIP to the MYC, triggering its degradation. A similar idea could be applied to MYCN, although it would be accelerated if there was an experimental structure of MYCN that has a suitable pocket for glue or PROTAC design. Until then, computational models of MYCN/MAX could provide a starting point.

## Conclusion

MYCN has a central role in neuroblastoma oncogenesis and malignancy, underscoring its value as a therapeutic target in high-risk disease. However, progress is limited by incomplete understanding of its interaction network, notably the scarcity of high-resolution structures for MYCN complexes. The set of protein interactions that have been structurally characterised only partially overlaps with those currently explored as potential drug targets. Future advances in therapies for MYCN-amplified neuroblastoma are therefore likely to focus on its network of protein complexes. Context-specific vulnerabilities will be exploited by combining disruption of selected protein–protein interactions with targeted degradation of key components within the MYCN interaction network.

## Unanswered questions

What is the full spectrum of MYCN-interacting proteins and what are their respective molecular mechanisms in driving neuroblastoma malignancy? What other physiological roles do these proteins play and will targeting them cause side effects?

Can we determine the precise binding sites and binding modes of interacting proteins on MYCN? Can we resolve the composition of different MYCN complexes, and understand the basis of competition or cooperation between interacting proteins?

Given the high homology between MYCN, MYC, and MYCL, how can drugs be designed to selectivity target conserved regions such as MB0 or MBII in MYCN?

Can the IDRs of MYCN be stabilised so as to create defined pockets for drug binding? Are there any novel approaches that exploit MYCN’s dynamic structure, turning a challenge into an opportunity?

What are the consequences of prolonged MYCN suppression on normal neuronal development and function? Could neurotoxicity be mitigated while maintaining therapeutic efficacy?

## Open Access

Open access for this article was enabled by the participation of the University of Leeds in an all-inclusive Read & Publish agreement with Portland Press and the Biochemical Society under an agreement with JISC.

## Abbreviations

AIH: AURKA interacting helix
AURKA: Aurora kinase A
BR: basic region
CRC: core regulatory circuit
E-box: enhancer-box
HAT: histone acetyltransferase complex
HDAC: histone deacetylase
HLH-LZ: helix-loop-helix-leucine-zipper
IDR: intrinsically disordered region
INSS: International Neuroblastoma Staging System
MB: MYC homology box
NCC: neural crest cell
NLS: nuclear localisation signal
PROTAC: proteolysis targeting chimera
TAD: transactivation domain
